# THE LONGITUDINAL RELATIONSHIPS BETWEEN SOCIAL ISOLATION AND HEALTH OUTCOMES: THE ROLE OF PHYSICAL FRAILTY

**DOI:** 10.1093/geroni/igac059.560

**Published:** 2022-12-20

**Authors:** Fereshteh Mehrabi, François Béland

**Affiliations:** University of Montreal, Montreal, Quebec, Canada; University of Montreal, Montreal, Quebec, Canada

## Abstract

Social isolation is a public health issue that is linked to poor health outcomes. However, the mechanisms underlying this association remain unclear. The main objective of this study was to explore whether changes in frailty moderated the relationship between changes in social isolation and changes in health outcomes over two years. We examined the mediating role of changes in frailty when the moderation hypothesis was not supported. A series of latent growth models (LGMs) were used to test our objectives using data from three waves of the FRéLE study among 1643 Canadian community-dwelling older adults aged 65 years and over. Missing data were handled by pattern mixture models with the assumption of missing not at random. We measured social isolation through social participation, social networks, and social support from different sources of social ties. We assessed frailty using the Fried frailty phenotype. Our moderation results revealed that high levels of changes in social participation, support from friends, nuclear, and extended family members, and social contacts with friends were associated with greater changes in cognitive and mental health among frail older adults with diminished physiological reserves compared to robust older adults. Additionally, changes in frailty mediated the effects of changes in social participation and social contacts and support from friends on changes in chronic conditions. This longitudinal study suggests that frailty moderated the relationships between social isolation and mental and cognitive health but not physical health. Overall, social support and strong friendship ties are key determinants of frail older adults’ health.

